# 1.F. Skills building seminar: Podcast 101 - Lessons learnt and how you can create your own unique experience

**DOI:** 10.1093/eurpub/ckac129.022

**Published:** 2022-10-25

**Authors:** Organised by: EUPHA-DH, Chair persons: Stefan Buttigieg (EUPHA-DH), Brian Li Han Wong (UK)

## Abstract

Podcasts are one of the fastest growing communication channels globally with more than 850,000 podcasts available globally through all the different podcast distributors. Furthermore, Health-related podcasts are gathering steam on their own front and herein lies the opportunity for public health and digital health professionals to embrace this channel and develop the necessary skills to start communicating their visions in a world that is plagued by misinformation and disinformation. This skills building workshop intends to cover the experience of three public health professionals in their involvement as co-hosts of three different podcasts that have their own voice with differences in their setup and availability of resources. Furthermore, the authors intend to cover the following aspects:

Getting started with your own podcasts

The typical setup for Podcast Recording

Promoting and growing your podcasts

Ensuring sustainability and consistency in your approach

Tips and Tricks for Podcast Success

**Key messages:**

• Podcasts are a unique opportunity to create a meaningful connection with a wide audience in a world plagued by misinformation and disinformation.

• Setting up a Podcast has gotten much less complicated and this workshop will provide the basic knowledge to get you started with tools that you already own.

